# Insulin/IGF-1 Signaling, including Class II/III PI3Ks, β-Arrestin and SGK-1, Is Required in *C. elegans* to Maintain Pharyngeal Muscle Performance during Starvation

**DOI:** 10.1371/journal.pone.0063851

**Published:** 2013-05-20

**Authors:** Donard S. Dwyer, Eric J. Aamodt

**Affiliations:** 1 Departments of Psychiatry and Pharmacology, Toxicology and Neuroscience, LSU Health Sciences Center, Shreveport, Louisiana, United States of America; 2 Department of Biochemistry and Molecular Biology, LSU Health Sciences Center, Shreveport, Louisiana, United States of America; Baylor College of Medicine, United States of America

## Abstract

In *C. elegans*, pharyngeal pumping is regulated by the presence of bacteria. In response to food deprivation, the pumping rate rapidly declines by about 50–60%, but then recovers gradually to baseline levels on food after 24 hr. We used this system to study the role of insulin/IGF-1 signaling (IIS) in the recovery of pharyngeal pumping during starvation. Mutant strains with reduced function in the insulin/IGF-1 receptor, DAF-2, various insulins (INS-1 and INS-18), and molecules that regulate insulin release (UNC-64 and NCA-1; NCA-2) failed to recover normal pumping rates after food deprivation. Similarly, reduction or loss of function in downstream signaling molecules (e.g., ARR-1, AKT-1, and SGK-1) and effectors (e.g., CCA-1 and UNC-68) impaired pumping recovery. Pharmacological studies with kinase and metabolic inhibitors implicated class II/III phosphatidylinositol 3-kinases (PI3Ks) and glucose metabolism in the recovery response. Interestingly, both over- and under-activity in IIS was associated with poorer recovery kinetics. Taken together, the data suggest that optimum levels of IIS are required to maintain high levels of pharyngeal pumping during starvation. This work may ultimately provide insights into the connections between IIS, nutritional status and sarcopenia, a hallmark feature of aging in muscle.

## Introduction

Insulin/insulin-like growth factor signaling (IIS) regulates various facets of growth and reproductive maturation, including energy metabolism, forkhead box O (FOXO) activity, and apoptosis [1]–[3]. In addition, this pathway controls the rate of development and aging in a wide range of organisms from *C. elegans* to man [4]–[7]. However, the role of IIS in aging is paradoxical. Reduced IIS is typically associated with increased longevity, i.e., a protective effect [4], [5], [7], [8]. On the other hand, insulin-like growth factor-1 (IGF-1) levels decrease as a function of aging and lead to a decline in muscle function [9]–[12]. When IIS activity is partially reduced in *C. elegans* temperature-sensitive *daf-2* (insulin/IGF-1 receptor) mutants by shifting the culture temperature from 15°C to 20°C, lifespan is significantly extended in all animals in the population. By contrast, when IIS is further reduced by growth at 25°C, roughly half of the animals die prematurely, whereas the other half lives much longer than wild-type controls [4]. This relationship has also been observed in *Drosophila* [5], [13]. Thus, reducing IIS can either lengthen or shorten lifespan depending on factors that have not been characterized. The findings in *C. elegans* are especially intriguing because the animals are genetically identical, yet die at very different ages.

The negative role of IIS in aging contrasts with its positive role in muscle function. Studies from the 1970’s demonstrated that insulin produced positive ionotropic effects on heart muscle [14], [15]. More broadly, IIS regulates energy metabolism, contractile protein expression and overall size of the heart [16]. In addition, IGF-1 levels decrease as a function of age, which contributes to loss of muscle mass and sarcopenia in the elderly population [10], [17], [18]. Insulin resistance frequently emerges with advanced age and is associated with impaired muscle function [19], [20]. By contrast, IIS blocks muscle atrophy leading to tissue hypertrophy, and overexpression of IGF-1 in muscle attenuates aging-related decline in function [21], [22]. Both insulin and IGF-1 regulate the mammalian target of rapamycin (mTOR) signaling pathway and stimulate protein synthesis [23], [24], which decreases in muscle with age [25], [26]. Finally, IIS directly affects the expression and activity of proteins critical for muscle function, including Ca^++^ channels, ryanodine receptors, and sarco-endoplasmic calcium-transport ATPases [27]–[30].

IIS is mediated through the binding of insulin and insulin-like ligands to their cognate cell surface receptors, followed by phosphorylation and association of insulin receptor substrate (IRS) proteins, and activation of phosphatidylinositol 3-kinase (PI3K), phosphatidylinositol-dependent kinase-1 (PDK-1) and Akt. In addition, there is receptor crosstalk between insulin/IGF-1 receptors and other signaling pathways including G proteins (Gα and Gβγ) and β-arrestin [31]–[33]. This co-activation of insulin/IGF-1 receptors and G proteins probably accounts for some of the positive effects of muscle activity, neuronal innervation, and β-adrenergic agonists on muscle mass and strength [21], [34]. Activation of PDK-1 leads to phosphorylation and activation of Akt, which phosphorylates downstream targets, including FOXO, glycogen synthase kinase 3 (GSK3), Bcl-2 antagonist of cell death, Akt substrate of 160 kDa; PDK-1 also phosphorylates kinases related to Akt, such as serum- and glucocorticoid-inducible kinase (SGK), p70 S6 kinase (S6K), and protein kinase C [2]. Akt phosphorylates ion channels and transporter proteins relevant for muscle function [30], [35], [36].

The studies described here were inspired by the paradoxical findings related to IIS in aging vs. muscle function. To investigate this paradox, we studied the role of this pathway in *C. elegans* in the recovery of pharyngeal pumping rate during starvation. Previously, Avery and Horvitz [37] showed that *C. elegans* reduces pumping by ∼ 50% upon acute removal from food (bacteria), but then recovers close to the baseline rate on food after 6–24 hr of food deprivation. Preliminary studies by our group revealed that the *C. elegans* insulin/IGF-1 receptor, DAF-2, and insulin molecules (e.g., INS-1) were required for recovery of pharyngeal pumping during starvation [38]. Here, we investigated recovery of pharyngeal pumping in various mutant strains, and in the absence or presence of inhibitors to clarify the role of IIS components in the response. These studies revealed that IIS maintains muscle function during starvation via downstream targets that regulate excitability, energy metabolism, and autophagy.

## Materials and Methods

### Strains and Culture Conditions

*C. elegans* strains were grown under standard conditions [39] on petri-plates with nematode growth medium (NGM) and OP50 *E.coli* as the food source. Strains were typically incubated at 15°C unless otherwise indicated.

The following strains were obtained from the Caenorhabditis Genetics Center: RB759 *akt-1(ok525),* GR1310 *akt-1(mg144),* RB660 *arr-1(ok401),* JD21 *cca-1(ad1650),* DR1566 *daf-2(m579),* DR1565 *daf-2(m596),* DR1942 *daf-2(e979),* CB1370 *daf-2(e1370),* DR1568 *daf-2(e1371),* DR1574 *daf-2(e1391),* CF1038 *daf-16(mu86),* DR1408 *daf-16(m26);age-1(m333),* RB712 *daf-18(ok480),* JT191 *daf-28(sa191),* VC1218 *ins-18(ok1672),* RB2552 *ins-31(ok3543),* GR1318 *pdk-1(mg142),* RB1813 *piki-1(ok2346)*, VC345 *sgk-1(ok538),* CB5 *unc-7(e5),* CB246 *unc-64(e246),* NM547 *unc-64(js21),* CB540 *unc-68(e540),* DR1089 *unc-77(e625)*, CB1068 *unc-79(e1068),* CB1272 *unc-80(e1272),* RB1494 *R09B5.11(ok1759),* XA7401 *R09B5.11(ok1759)* (RB1494 outcrossed 4x by A. Wolstenholme; University of Bath). Most of these strains have been back-crossed several times to the wild-type background. The exceptions are: *akt-1(ok525)*, *arr-1*, *daf-18*, *ins-1*, *ins-31*, *piki-1* and *sgk-1*. The *FXO1888 ins-1(tm1888)* strain was obtained from the National Bioresource Project, Japan (Dr. Shohei Mitani). The double knockout strain, *nca-1(gk9);nca-2(gk5)* [40], was kindly provided by Dr. Mei Zhang (University of Toronto). We produced the *ins-1(tm1888);ins-18(ok1672)* strain by standard breeding methods, and confirmed gene knockout by polymerase chain reaction with specific oligonucleotide primers for *ins-1* (left – TGTACTGGTTTCGTCA-AGTTTACAGA and right – AATTATCGTCCTGATTGCAGCAGA) and *ins-18* (left – TGGTCCACCGACTTTTCAT and right – AAATTGGGGCACAGTAGGCAAGA).

### Recovery of Pharyngeal Pumping

For these studies, we first evaluated the pharyngeal pumping rate on food by counting the number of pumps made during a 30 sec observation period and deriving the pumps per minute (PPM) as described previously [41]. We obtained data from young adults, i.e., worms 1–2 days past the L4 stage, at room temperature after growth at 15°C (or 25°C in the case of temperature-sensitive strains, e.g., *daf-2(e1391)*). Pumping (movement of the corpus and grinder) was examined with a Leica MZ12 microscope at 100x magnification. After counting pumping on food, worms were then picked off food to a bacteria-free part of the plate and allowed to crawl for 5–10 sec prior to transfer to 100 mm NGM plates without food to prevent transfer of bacteria. After 2, 3.5, 6 and 24 hr on the bacteria-free plates, we once again measured the rate of pharyngeal pumping. For most of these experiments, data were averaged from 20 animals per group, which is standard for the field (e.g., see Steger et al. [42]).

### Inhibitor Studies

The PI3K inhibitor, LY294002, and Ca^++^ channel antagonists, mibefradil, nifedipine and flunarizine, were obtained from Sigma-Aldrich (St. Louis, MO). They were dissolved in DMSO and diluted in weak (10^−4^ M) acetic acid for addition to plates. Control plates received identical amounts of DMSO-dilute acetic acid. Diluted compounds (200 µl final volume) were added to 60 mm NGM plates with bacteria and allowed to dry overnight. Animals were then transferred with a thin platinum wire to the plates containing inhibitor or vehicle, and then remained on these plates for 1.5 hr before transfer to bacteria-free test plates. Pharyngeal pumping was assayed periodically as described above. The final concentrations of inhibitors used here were: LY294002 (150 µM), mibefradil (50 µM), nifedipine (50 µM) and flunarizine (100 µM). These concentrations were chosen on the basis of relevant literature in the case of LY294002 [43] or pilot studies with the channel antagonists.

### Effect of 2-deoxyglucose (2-DOG)

2-DOG is taken up by cells via glucose transporters (GLUTs), and inhibits further cellular glucose metabolism. Long-term exposure of *C. elegans* to 5 mM concentrations is optimal for extending lifespan [44]. For our experiments, 2-DOG was added to NGM plates with bacteria at a final concentration of 5 mM in water; control plates received the same volume of water. After the liquid dried, animals were transferred to the plates and incubated overnight (∼16–20 hr) at 15°C. Animals were then picked to plates without bacteria, and pumping was measured as before.

### Statistical Analysis

To determine statistical significance of differences between groups, we performed repeated-measures analysis of variance with the package from OpenStat (provided by Dr. William Miller, online). A post hoc Newman-Keuls test was run to determine the level of significance for between-group comparisons. To preserve clarity in graphs with multiple plots, we have depicted the 99% confidence intervals for the control data. Group means lying outside this region (vertical bars) are significantly different from the control at the p<0.01 level. In other cases, significance levels are indicated with asterisks as described in the text, table, and figure legends.

## Results

### Insulin Receptor Required for Recovery of Pharyngeal Pumping

**Figure 1:**
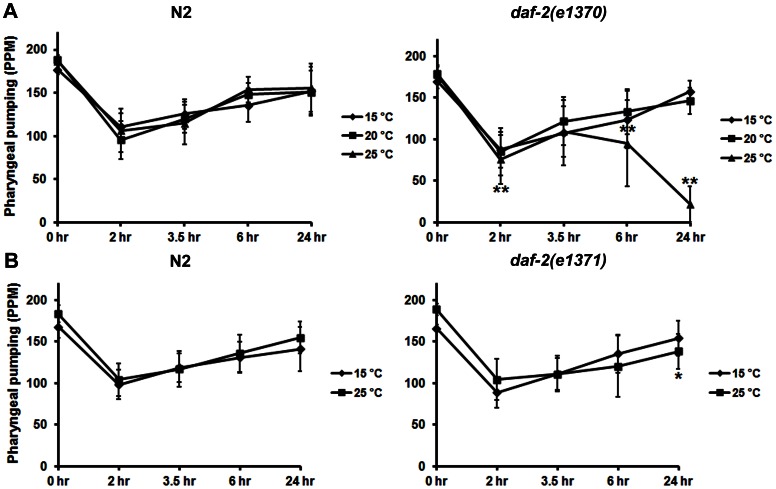
Reduction of function in DAF-2 prevents recovery of pharyngeal pumping. Young adult animals (15 per group) were grown overnight at the temperatures indicated (♦ 15°C; ▪ 20°C, or ▴ 25°C) and pharyngeal pumping was then evaluated on food (0 hr time point) and 2, 3.5, 6 and 24 hr after removal from bacteria. The data are expressed as the average pumps per minute (PPM). Standard deviations are represented by the error bars. Significant differences from the N2 controls are indicated by asterisks, *p<0.05; **p<0.01 as determined by ANOVA and a Newman-Keuls test. Results are shown for temperature-sensitive strains, A) *daf-2(e1370)* and B) *daf-2(e1371)*.

These studies were inspired by our interest in how IIS regulates appetite and feeding [45]. Previously, Avery and Horvitz [37] reported that acute starvation caused a rapid reduction in the rate of pharyngeal pumping followed by a gradual recovery of fast pumping as a function of time off food. This system appeared to offer the chance to explore whether IIS regulates the rate of pharyngeal pumping, which strongly correlates with aging in *C. elegans* [46], [47]. As a first step toward this goal, we evaluated recovery of pharyngeal pumping in two strains with temperature-sensitive defects in DAF-2 function. For these experiments, the various *daf-2* strains and N2 controls were shifted to 25°C for 16–20 hr to reduce DAF-2 activity prior to removal from food. Because we focused on young adults in these studies, the timing and duration of the temperature shift rule out chronic or developmental effects of DAF-2 deficiency as contributing factors in the response. Recovery of pharyngeal pumping in the absence of food was also carried out at 25°C. As seen in Fig. 1A, the *daf-2(e1370)* strain failed to recover normal pumping rates after 6–24 hr of food deprivation when grown at the restrictive temperature. By contrast, the *daf-2(e1371)* strain showed a modest (but significant) decrease in pumping compared to N2 controls only after 24 hr of starvation when shifted to 25°C (Fig. 1B). Gems et al. [48] have previously characterized various *daf-2* alleles with respect to their degree of severity on different biological measures (e.g., thermotolerance and brood size). *daf-2(e1370)* is classified as a more severe phenotype (class 2C) than *daf-2(e1371)* (class 1A), in keeping with our results. Moreover, Gems et al. [48] reported that pharyngeal pumping declines with age in relation to the severity of the *daf-2* allele; this defective pumping is evident much earlier with starvation.

**Figure 2:**
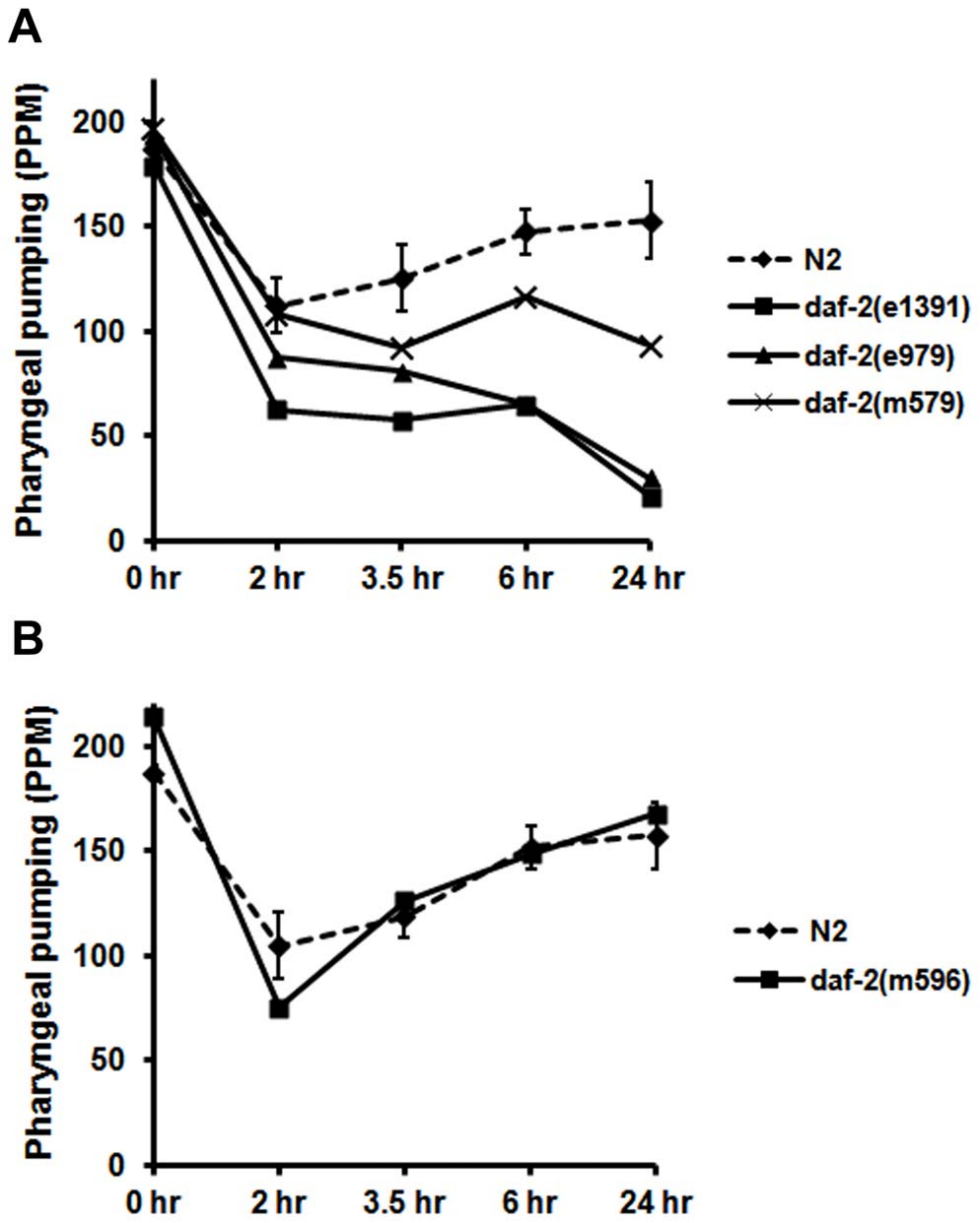
Severity of *daf-2* allele affects recovery of pharyngeal pumping during starvation. Young adults (20 per group) from the various strains indicated in A) & B) were grown overnight at 25°C and pharyngeal pumping was measured as described above. To simplify the figure, we have only shown the 99% confidence intervals around the means of the N2 control groups. Experimental group means lying outside these intervals are significantly different from the controls with a p value <0.01.

In view of these data, we evaluated additional *daf-2* strains that were shifted to 25°C as described above for recovery of pharyngeal pumping following food deprivation. The results in Fig. 2A confirm that functional knockout of DAF-2 inhibits recovery of pharyngeal pumping during food deprivation. Furthermore, there was a strong correlation between the severity of the *daf-2* alleles and the degree of recovery such that *e1391* and *e979*, which are both class 2D, pumped at much slower rates than the class 2B allele, *m579*. Interestingly, the *daf-2(m596)* strain showed a wild-type recovery profile (Fig. 2B) despite being a class 2A allele with multiple other defects, including reduced adult mobility, smaller brood size, and larval arrest at 25°C. This finding will be examined in more detail in the Discussion section. However, the location of the *m596* mutation in the DAF-2 protein suggested differences in ligand binding might account for our observations.

### Identification of Insulins Involved in Pumping Recovery

**Figure 3:**
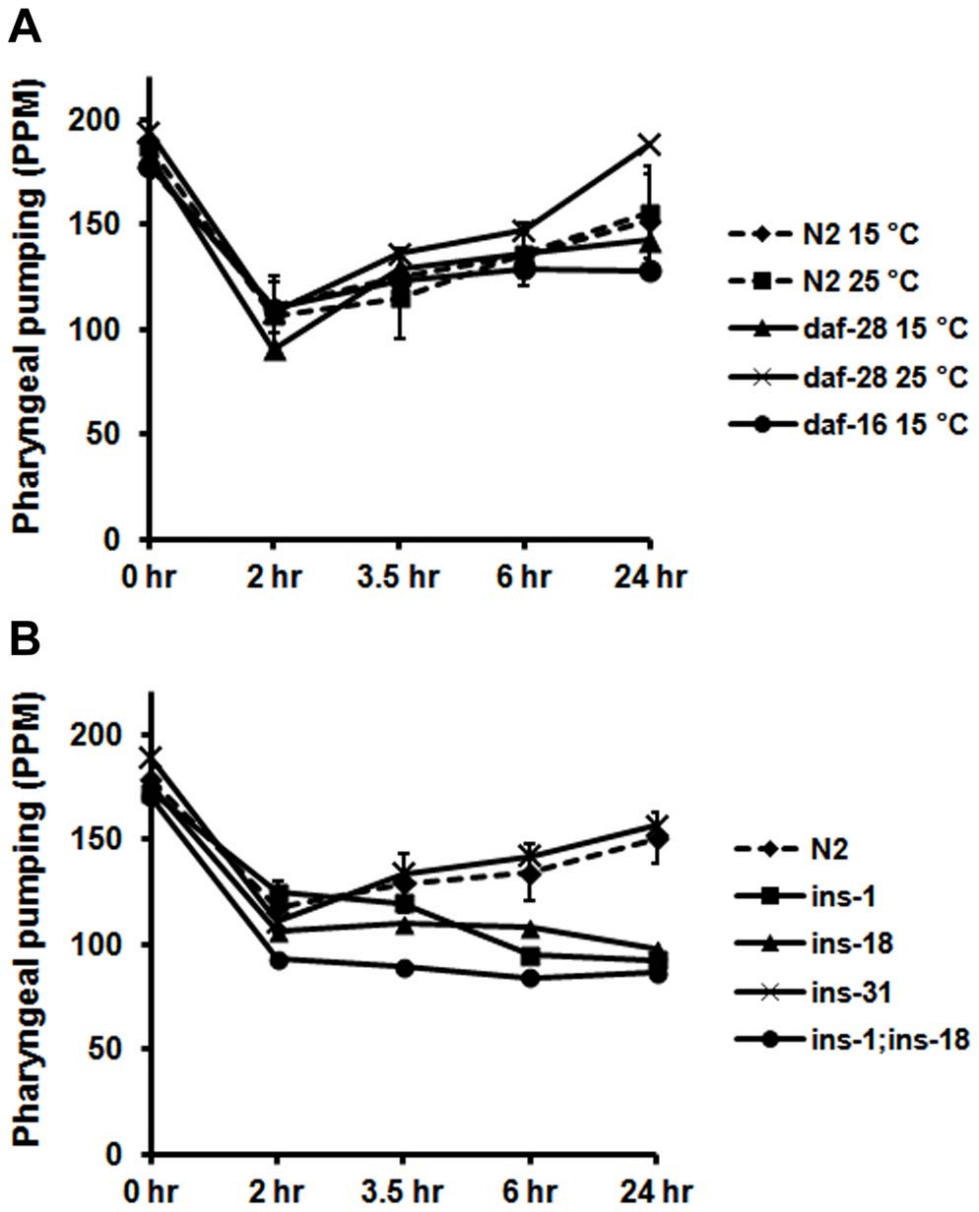
Identification of insulins involved in pumping recovery. A) Young adults were grown overnight at the temperatures indicated and recovery of pharyngeal pumping was assayed as before. Pumping in the *daf-28* strain (25°C) at the 3.5 and 24 hr time points was significantly (p<0.01) faster than the control. B) For these experiments, young adults from the various strains were grown at 15°C (the standard condition) and recovery of pumping during starvation was measured as before. The 99% confidence intervals around the control means have been depicted here.

To explore the contribution of DAF-2 ligands in the response, we evaluated recovery of pharyngeal pumping in a strain, *daf-28(sa191)*, with a temperature-dependent defect in the activity of DAF-28 [49], one of the major insulin molecules of *C. elegans*. In addition, we examined pumping recovery in the *daf-16(mu86)* null strain to determine whether this well-known target of IIS mediated the effects of DAF-2 in our system. As shown in Fig. 3A, functional reduction of DAF-28 at 25°C did not impede recovery of pharyngeal pumping. In fact, the pumping rate at 24 hr was significantly faster than in the N2 controls, or in *daf-28(sa191)* animals grown at the permissive temperature, indicating that DAF-28 insulin may have a slight inhibitory effect on pumping recovery. The lack of effect of *daf-16* (Fig. 3A) suggests that DAF-16-mediated gene expression is not required for early recovery of pumping. Thus, the effects of IIS on pumping appear to be mediated by acute changes in signaling pathways rather than by longer-term changes in gene regulation.

*C. elegans* has 40 separate insulin molecules that might constitute the ligand for DAF-2 in this response [50], [51]. INS-1 and INS-18 appeared relevant because these proteins are expressed in the pharynx [50]. Knockout strains lacking *ins-1* and *ins-18* do not show full recovery of pharyngeal pumping during starvation (Fig. 3B). Although the impairment is not as severe as in the *daf-2(e1391)* strain, the data are highly significant. By contrast, the *ins-31* strain recovered with the same profile as the wild-type control. We derived the double knockout strain, *ins-1;ins-18*, and examined its behavior in the assay (Fig. 3B). The double knockout strain showed similar behavior as the single knockout strains. Because *daf-2(e1391)* is affected to a greater degree than *ins-1;ins-18*, we believe that additional insulin molecules must be involved in the response.

### Effect of Mutations that Interfere with Insulin Secretion

**Figure 4:**
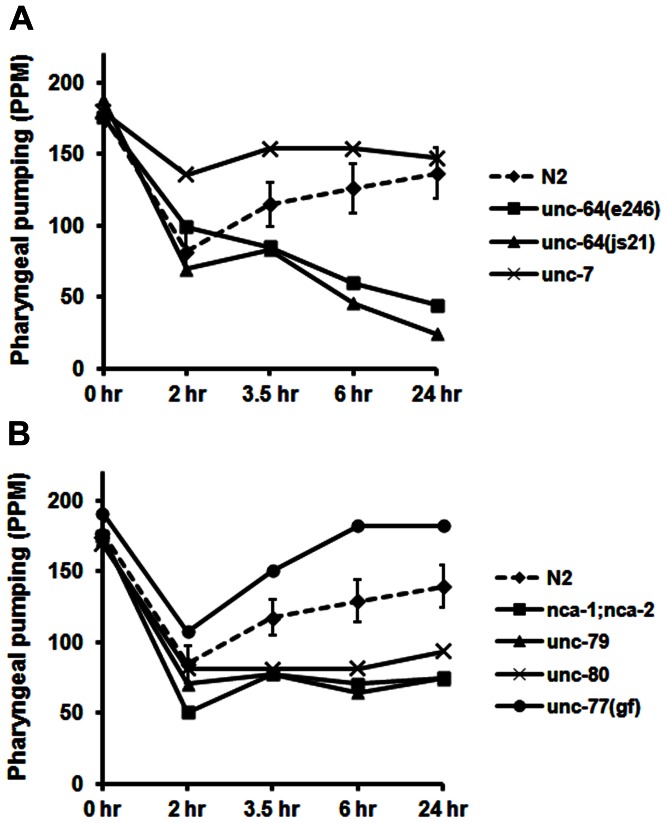
Effect of mutations in genes that potentially regulate insulin/neuropeptide release. A) & B) Young adults (20 per group) from the various strains indicated in the figure were evaluated for recovery of pumping during food deprivation under standard conditions. The 99% confidence intervals for the control data are indicated as before.

A number of proteins regulate the secretion of insulin in *C. elegans* and humans, including UNC-64 (syntaxin), and the Na^+^ leak current protein (NALCN) [52]. We reasoned that if insulin is involved in restoring the rate of pharyngeal pumping, then strains with mutations in genes encoding these proteins should be defective in pumping recovery. Strains with two different loss-of-function (*lf*) alleles of *unc-64* pumped at typical rates on food, but failed to recover normal pumping in response to food deprivation (Fig. 4A). The *nca-1;nca-2* strain is functionally deficient in both NALCN proteins [40]. Although *nca-1* has an original designation as *unc-77*, we have used the descriptive name for the gene here. The *nca-1;nca-2* strain pumped normally on bacteria, but was unable to recover normal pumping rates after 2–24 hr of starvation (Fig. 4B). UNC-79 and UNC-80 appear to direct NALCN to the plasma membrane or act as subunits of this ion channel because loss of these proteins abolishes cell surface expression [53]–[55]. *unc-79*, *unc-80* and *nca-1;nca-2* exhibit identical movement defects, which is consistent with these genes being required for the same physiological functions [53], [56]. Not surprisingly, *unc-79* and *unc-80* showed the same profile as *nca-1;nca-2* in our assay (Fig. 4B). By contrast, *unc-77(e625)* is a gain-of-function (*gf*) allele of *nca-1*. As seen in Fig. 4B, this strain recovered pharyngeal pumping at a significantly accelerated rate compared to the wild-type control.

The next experiment examined pumping recovery in the *unc-7* strain, which is an *lf* allele of a gene coding for a gap junction channel protein (innexin). In various behavioral assays, *unc-7* mutations reverse the phenotype of the *unc-79* and *unc-80* double mutants [40], [53], [56]. Consistent with these observations, loss of *unc-7* resulted in faster recovery of pharyngeal pumping, similar to *unc-77(gf)*, although the positive effects on recovery were diminished by the 24-hr time point (Fig. 4A). The role of *unc-7* in this response is likely to be complex, and will require further investigation. Nevertheless, the data suggest that regulation of insulin (and perhaps other neuropeptides) release is somehow involved.

### IIS Downstream Pathway Regulates Pumping Recovery

**Figure 5:**
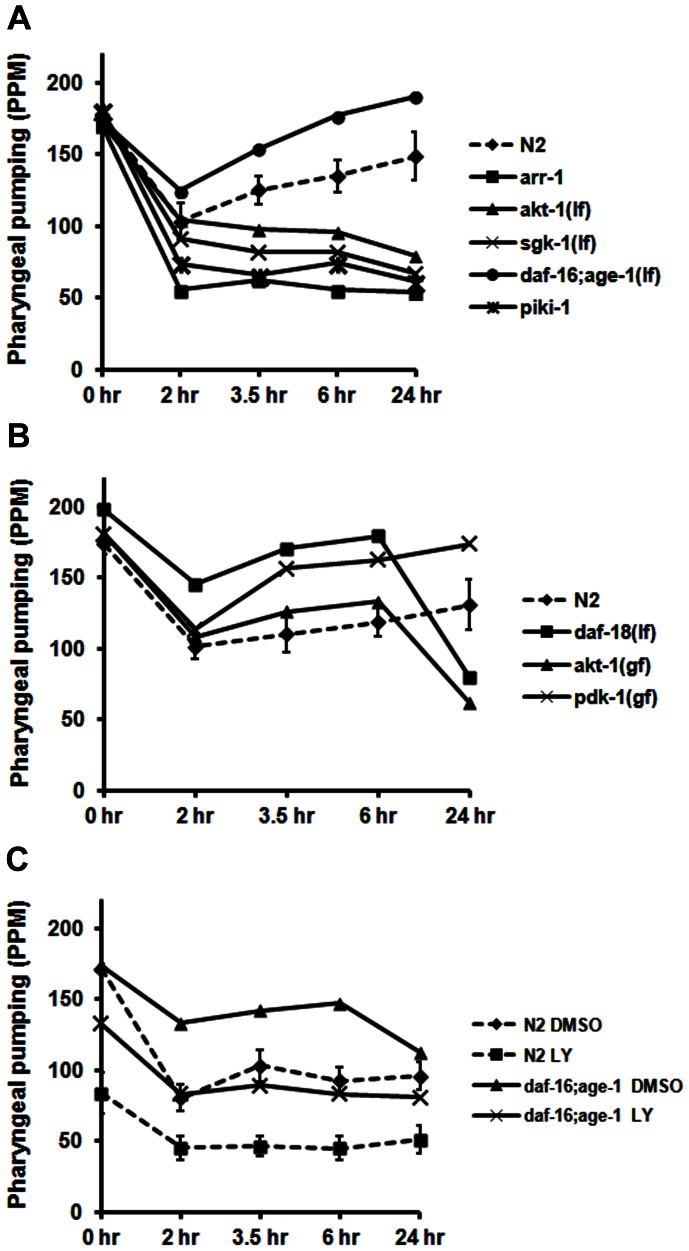
Effect of mutations in genes encoding signaling molecules downstream of DAF-2 on the recovery response. A) & B) The strains listed here were evaluated under standard conditions described above; *gf* and *lf* refer to gain-of-function and loss-of-function mutations, respectively. C) For these experiments, young adults (20 per group) from the N2 and *daf-16; age-1* strains were incubated on plates with food and either DMSO (0.8%; vehicle control) or LY294002 (LY; 150 µM) for 1.5 hr at 15°C prior to food deprivation and evaluation of pharyngeal pumping. The 0-hr data point represents pumping on food in the presence of either DMSO or LY294002. The 99% confidence intervals for the control data are depicted for comparison.

Binding of insulin to the DAF-2 receptor activates downstream signaling molecules such as PI3K, PDK-1 and Akt. We explored the contributions of these proteins to recovery of pharyngeal pumping by examining the response of various *lf* and *gf* strains. The first two *lf* strains tested, *akt-1(ok525)* and *sgk-1(ok538)*, failed to recover pharyngeal pumping after 3.5–24 hr of food deprivation (Fig. 5A). Interestingly, the *arr-1(ok401)* strain, a null allele of the single *C. elegans* β-arrestin gene, also showed a significantly diminished rate of pharyngeal pumping over the course of the assay (Fig. 5A). β-arrestin has previously been linked to IIS activation [33], [57], and may play a similar role in our system. As might be expected based on these results, strains with *gf* alleles of Akt [*akt-1(mg144)*] and PDK-1 [*pdk-1(mg142)*] recovered pumping significantly faster than the N2 strain (Fig. 5B). The *daf-18(ok480)* strain is a null allele of the PTEN phosphatase, which dephosphorylates phosphatidylinositol 3,4-phosphate (PI(3,4)P_2_) and phosphatidylinositol 3,4,5-phosphate (PI(3,4,5)P_3_) to terminate signaling via class I/II PI3Ks. Knockout of *daf-18* extends the actions of PI(3,4)P2 and PI(3,4,5)P_3_ and increases Akt/SGK signaling. As seen in Fig. 5B, the *daf-18* strain rapidly recovers pharyngeal pumping over the first 2–6 hr off food, but then drops off dramatically after 24 hr of starvation. The *akt-1(gf)* strain (Fig. 5B) showed an identical profile as *daf-18* (although shifted down on the y-axis), whereas the *pdk-1(gf)* strain maintained faster recovery throughout the time period examined. Thus, increased activity of AKT-1 in the (*gf*) strain (and in *daf-18*) promotes the initial recovery of pumping presumably through its actions on downstream effectors such as Ca^++^ channels (see next section). However, sustained activation of AKT-1 during food deprivation may inhibit autophagy, which is required to maintain pumping after 24 hr off bacteria.

The data with *akt-1* and *sgk-1* pointed to a potential role for PI3K in the recovery of pharyngeal pumping. To examine this possibility, we used the PI3K inhibitor, LY294002, to block the IIS pathway at the level of AGE-1. As anticipated, LY294002 inhibited recovery of pharyngeal pumping, confirming a role for PI3K in this response (Fig. 5C). Interestingly, LY294002 also significantly reduced basal rates of pharyngeal pumping on food (Fig. 5C). To establish whether AGE-1 was indeed the target of LY294002, we evaluated recovery of pumping in a strain with a null allele of *age-1*. Most *age-1* alleles have modest to moderate *lf* phenotypes, whereas worms that are null for *age-1* arrest as constitutive dauers unless the mutations are balanced by knocking out *daf-16* [58]. Fig. 5A reveals that the *daf-16(m26);age-1(m333)* strain rapidly recovered pharyngeal pumping during starvation at a rate that was surprisingly faster than normal. It is unlikely that this effect is due to the *daf-16(m26)* allele because *daf-16(mu86)* did not affect pumping recovery (Fig. 3A). We then tested whether LY294002 inhibited pumping recovery in this *age-1* null strain. As seen in Fig. 5C, LY294002 reduced pharyngeal pumping on food, and prevented recovery of pumping off food, despite the absence of class I PI3K in the *daf-16(m26);age-1(m333)* strain. Taken together, these findings suggest that AGE-1 normally inhibits recovery of pharyngeal pumping in response to starvation, and that LY294002 targets a different class of PI3Ks (II and/or III) to inhibit the response. To evaluate contributions by class II PI3K, we examined pumping recovery in the *piki-1(ok2346)* strain, which has a central deletion mutation, and is associated with accumulation of cell corpses. As seen in Fig. 5A, *piki-1* is required for recovery of pharyngeal pumping, which supports the role of class II PI3K in the response.

### Role of IIS Effector Proteins

How does IIS ultimately affect the recovery of pharyngeal pumping? Candidate downstream targets of this pathway include Ca^++^ channels and glucose transporters (GLUTs). We examined the amino acid sequences of a number of candidate proteins to identify possible Akt/SGK phosphorylation sites – RXRXX**S/T**Hy, where X is any amino acid and Hy represents a hydrophobic or bulky residue. Table 1 summarizes the results of this sequence analysis. The Akt motif is found in Ca^++^ channel proteins, CCA-1 and UNC-2, the ryanodine receptor, UNC-68, the glucose transporter encoded by R09B5.11, and intriguingly, NCA-1, UNC-80 and UNC-79. Additional studies will be needed to confirm phosphorylation of these sites by Akt/SGK; however, these observations motivated us to evaluate recovery of pumping in strains with mutations in *cca-1*, *unc-68*, and *R09B5.11(ok1759)*, a *C. elegans* ortholog of GLUT1/GLUT4. Because human GLUT4 also has a putative Akt phosphorylation site, whereas other human GLUTs do not, we favor the idea that R09B5.11 is the *C. elegans* equivalent of GLUT4, which is regulated by insulin signaling.

**Table 1:** Phosphorylation Motifs in Potential Targets of Akt/SGK^a^.

Protein	Amino acid sequence						
Consensus	R	X	R	X	X	S/T	Hy
CCA-1 (1)	R	A	R	T	N	S	A
CCA-1 (2)	R	P	R	S	R	S	H
UNC-2 (1)	R	Y	R	T	E	S	P
UNC-2 (2)	R	N	R	T	N	T	M
R09B5.11	R	K	R	I	C	S	
hGLUT4	R	E	R	P	L	S	L
UNC-68 (1)	R	Y	R	I	E	S	L
UNC-68 (2)	R	L	R	D	S	S	N
NCA-1	R	A	R	E	A	T	T
UNC-79	R	L	R	E	F	T	D
UNC-80	R	H	R	F	V	S	F

a Amino acid sequences of motifs that conform to the consensus phosphorylation site of Akt/SGK. The sequences are derived from the *C. elegans* proteins with the exception of hGLUT4, which is human GLUT4. The single letter code has been used here. X is any amino acid, whereas Hy refers to a hydrophobic or bulky residue.

**Figure 6:**
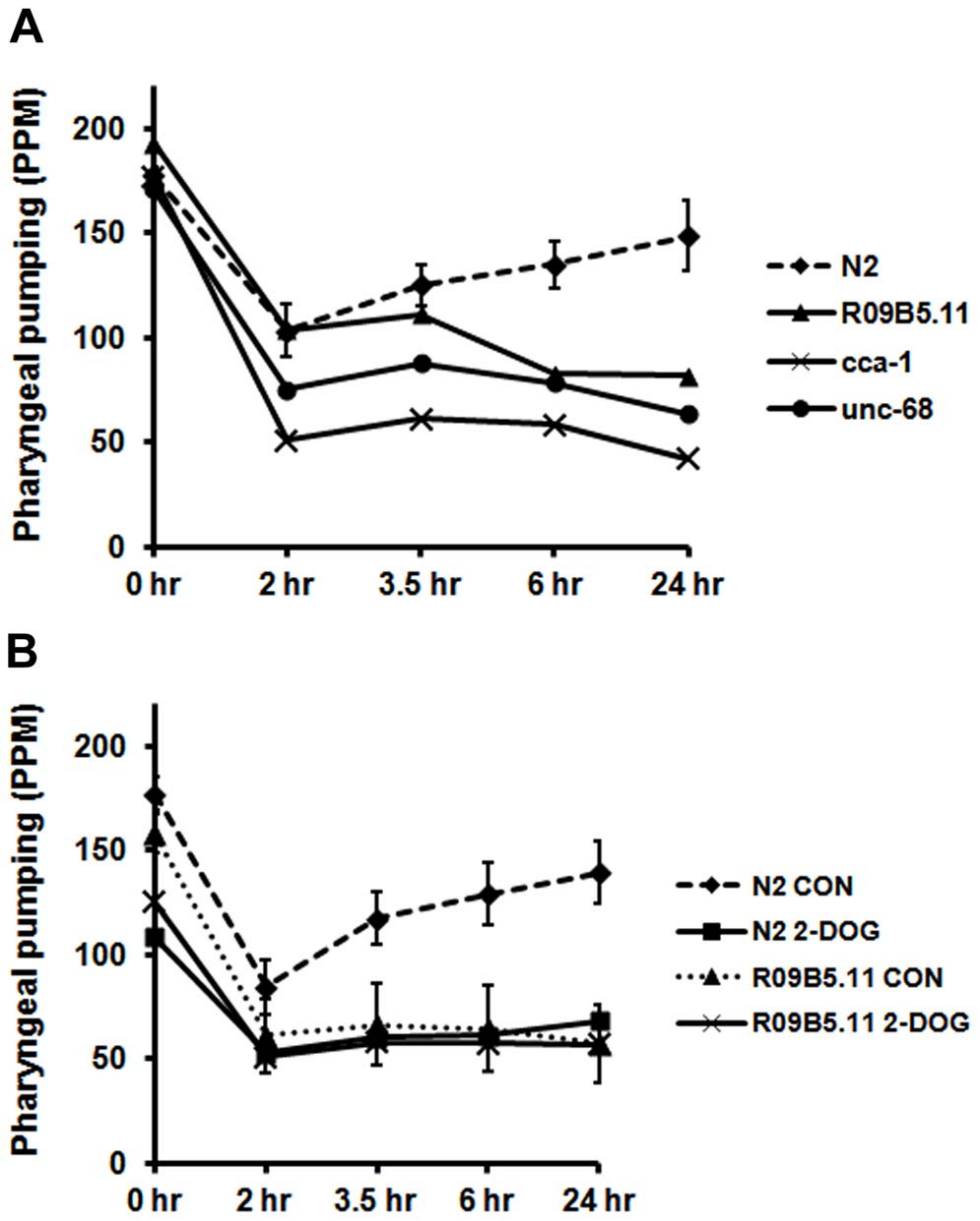
Role of potential downstream targets of IIS in recovery of pharyngeal pumping. A) The strains shown here were evaluated under standard conditions described above. For *R09B5.11(ok1759)* experiments, the XA7401 strain was used. B) Young adults (20 per group) from the N2 or XA7401(*R09B5.11*) strains were grown overnight on plates with food and 2-deoxyglucose (2-DOG; 5 mM final concentration) or dilute acetic acid (control; CON). The next day, they were evaluated for recovery of pharyngeal pumping off food. The 99% confidence interval for control means (N2 or *R09B5.11*) are shown as before.

The results in Fig. 6A show that *cca-1* and *unc-68* pump at relatively normal rates on food, but are significantly impaired in recovery of pharyngeal pumping upon starvation. UNC-2 is clearly important for pharyngeal pumping; however, *unc-2* strains pump very slowly even on food [59], which was the reason to forego testing of these mutants in our system.

In addition, we pursued a pharmacological approach to the role of Ca^++^ channels in pumping recovery. In the initial experiments, we evaluated the effects of flunarizine (a broad spectrum Ca^++^ channel inhibitor), mibefradil (mixed T-type and L-type inhibitor) and nifedipine (L-type channel blocker) on N2 animals. CCA-1 is a T-type channel of *C. elegans*, whereas UNC-2 is a P/Q-type channel. The results in Table 2 confirm that inhibition of Ca^++^ channels interferes with basal pharyngeal pumping and recovery of pumping during food deprivation. It is noteworthy that mibefradil further inhibited pumping recovery in the *cca-1* strain, which suggests contributions by more than one Ca^++^ channel type to the response. We also observed that mibefradil produced an additional reduction in pumping in the *ins-1;ins-18* strain (Table 2), which suggests actions via parallel pathways and/or that mibefradil affects the synthesis/release of additional insulins involved in the response.

**Table 2:** Effect of Ca^++^ Channel Inhibitors on Recovery of Pharyngeal Pumping^a^.

Strain	Condition	On food	Off food	Off food	Off food	Off food
			2 hr	3.5 hr	6 hr	24 hr
				(PPM ± S.D.)		
N2	DMSO	177±12.8	84.6±20.2	117.2±19.4	129.2±23	139.4±23.6
N2	FLN	29±17.6**	34.8±15.8**	36±18**	26±16.6**	10.4±20**
N2	MIB	82.4±43**	23±20.8**	26.8±24.6**	21.4±16.8**	24.4±20.3**
N2	NIF	130.4±21.8**	73.8±30.4	82.6±40.6*	70±41.6**	49.4±39.4**
*cca-1*	DMSO	129.8±23.6	34±24.2	50±26	43.4±26.4	48.8±22.4
*cca-1*	MIB	71±48.2**	14.2±14	29±26.2	25±21.8	22.1±20**
*ins-1;ins-18*	DMSO	164.2±14.2	65.6±28.6	81±34	85±28	90±34.6
*ins-1;ins-18*	MIB	96.2±38.4**	43.4±28.4	52±28*	57.6±24.6*	63.2±30.2*

a Ca^++^ channel inhibitors were evaluated for their effects on recovery of pharyngeal pumping in the strains shown here. Young adults (20 per group) were incubated on flunarizine (FLN; 100 µM), mibefradil (MIB; 50 µM), nifedipine (NIF; 50 µM) or DMSO (0.4%, control) for 1.5 hr prior to food deprivation. The data represent the average ± the standard deviation (S.D.). Asterisks indicate significant differences compared to the corresponding DMSO control group:

* p<0.05,

** p<0.01.

Next, we evaluated two *R09B5.11(ok1759)* strains in our assay: RB1494, which is the parent strain, and XA7401, which has been backcrossed 4 times. Both strains failed to recover normal pharyngeal pumping, but the deficit was mainly evident at the 6 and 24-hr time points (Fig. 6A; data not shown for RB1494).

The findings with *R09B5.11(ok1759)* suggested that maintaining adequate levels of glucose during starvation is critical for full recovery of pharyngeal pumping. To test this possibility, we grew worms on 2-deoxyglucose (2-DOG) overnight to inhibit oxidation of glucose as reported by Schulz et al. [44]. Wild-type worms grown on 2-DOG showed a modest, but significant, decrease in pumping on food, and pumped at significantly decreased rates throughout the assay period (Fig. 6B). Overall, the effect of 2-DOG on the N2 strain was similar to the effect of knocking out the *R09B5.11* GLUT in the XA7401 strain. Interestingly, 2-DOG modestly reduced pumping of the XA7401 strain on food, but did not further inhibit pumping recovery in this strain (Fig. 6B). It would appear that additional GLUTs contribute to basal glucose transport during feeding, but that R09B5.11 may be the major transporter responsible for glucose uptake during food deprivation in the cells required for pumping recovery.

### Combined Effects of LY294002 and Gene Mutations on Pumping Recovery

Because LY294002 inhibited pumping recovery in the N2 strain, we reasoned that it might produce additive effects with some of the mutations that alter this response. For these experiments, animals from the strains of interest were incubated on plates with LY294002 (150 µM) or an equivalent concentration of DMSO (control) for 1.5 hr prior to transfer to bacteria-free plates that contained either LY294002 or DMSO to maintain the initial drug conditions throughout the assay. The results are summarized in Table 3, and several findings stand out. First, LY294002 reduces basal pharyngeal pumping on food in all of the strains tested by an overall average of 40%. Secondly, DMSO by itself at a final concentration of 0.8% reduced recovery of pharyngeal pumping in *daf-2(e1391)*, *unc-64(e246)*, *ins-1;ins-18*, XA7401, and N2 compared with dilute acetic acid as the solvent (contrast DMSO data in Table 3 with corresponding dilute acetic acid data in the figures). The effect of DMSO on *daf-2(e1391)* was quite remarkable. The animals failed to initiate movement after a 30 min exposure to DMSO (and food deprivation), and pharyngeal pumping was essentially abolished. However, the animals vigorously escaped when touched on the tail with a platinum wire demonstrating that they are not paralyzed under these conditions. Moreover, they fully recovered movement, growth and egg-laying after return to food, even after 24 hr on DMSO. Thirdly, LY294002 significantly decreased pharyngeal pumping more than genetic mutation alone in the *ins-1;ins-18*, *unc-64(e246)*, *nca-1;nca-2*, and *cca-1* strains. Although DMSO has modest effects on pumping recovery, possibly due to its actions on insulin signaling pathways (see Discussion), LY294002 significantly suppressed recovery in these strains more than DMSO alone. These data suggest that LY294002 affects additional pathways (e.g., class III PI3Ks) besides those impacted by the mutations studied.

**Table 3:** Inhibition of PI3Ks Affects Recovery of Pharyngeal Pumping^a^.

Strain	Condition	On food	Off food	Off food	Off food	Off food
			2 hr	3.5 hr	6 hr	24 hr
				(PPM ± S.D.)		
N2	DMSO	171.6±11.2	80.6±29.8	103.2±35.8	92.6±29.2	96±31.2
N2	LY	83.6±44.2**	45.4±26.4**	46.4±22.4**	45±38.6**	50.8±30.8**
*daf-2(e1391)* #	DMSO	181.1±10	9.0±20.8	4.4±12.8	7.6±22.4	8.2±26.4
*daf-2(e1391)* #	LY	103.8±47.2**	1.4±3.8	2.2±4.2	0.2±0.6	0.2±0.6
*ins-1;ins-18*	DMSO	131.2±20.4	62.4±21.8	59.8±27.2	55.4±25.6	50.8±30
*ins-1;ins-18*	LY	106.6±14.8**	28.2±24.6**	36.6±23.2*	44.4±23	44.8±27.8
*unc-64(e246)*	DMSO	104.2±14.4	53.2±28	27±16	31.8±21.2	23.2±16.6
*unc-64(e246)*	LY	85±19.6**	15.8±12**	17.2±11.6	19.8±11	9.4±10.8
*nca1;nca-2*	DMSO	162.8±12.4	70.6±29.6	78.6±27.6	73.8±31.2	65±30.1
*nca1;nca-2*	LY	99.8±36.2**	38.6±26.2**	53.8±32.4*	34±29.6**	45.4±27.4
*arr-1(ok401)*	DMSO	178.6±9.2	67.4±24.2	78.4±28.4	70.4±34.4	73.2±25.6
*arr-1(ok401)*	LY	68.4±36.2**	44.2±30.6	71±32	63±23.4	62.8±34.8
*cca-1(ad1650)*	DMSO	117.8±14.8	44.4±19.4	42.4±25.4	32.2±21	43.2±22.8
*cca-1(ad1650)*	LY	68.6±34.6**	22±15.4*	25.8±15.8	27.8±19.8	28.2±20.4
*XA7401*	DMSO	145.2±22.8	55.4±30.8	58.8±31.6	57.8±29.6	58.8±37.6
*XA7401*	LY	90±26.8**	38±27.6	42.2±30.4	44.8±35.2	54.2±36.8
*pdk-1(mg142)*	DMSO	177±16.8	85.8±40.6	105.2±40.2	105.4±33.2	118.2±38
*pdk-1(mg142)*	LY	89.8±60.2**	67.4±28.8	80.2±36.6	89.6±38.6	117.4±46

a Young adults (20 per group) were grown on plates treated with LY294002 (LY; 150 µM) or an equivalent amount of DMSO diluent for 1.5 hr before transfer off food. Pharyngeal pumping was measured periodically as before and the means and S.D. are summarized here. Significant differences from the corresponding DMSO control group have been indicated with asterisks:

* p<0.05,

** p<0.01.

# The *daf-2(e1391)* strain was grown at 25°C overnight prior to testing, and during food deprivation.

## Discussion

These studies revealed a pivotal role for IIS in the recovery and maintenance of high rates of pharyngeal pumping in response to starvation. The pumping rate rapidly declines within 30 min after removal from food, and then begins to recover 1–2 hr later. Already at this early stage, some of the mutant strains (e.g., *daf-2(e1391)*, *arr-1* and *sgk-1*) show impaired recovery of pharyngeal pumping compared to the controls. The data suggest an early recovery phase mediated by insulin-DAF-2-class II/III PI3Ks-AKT-1, and downstream targets (e.g. Ca^++^ channels), and a later phase involving DAF-2-PIKI-1-PDK-1 and possibly the autophagy pathway, which is suppressed by excessive PI(3,4,5)P_3_ and AKT-1 signaling.

**Figure 7:**
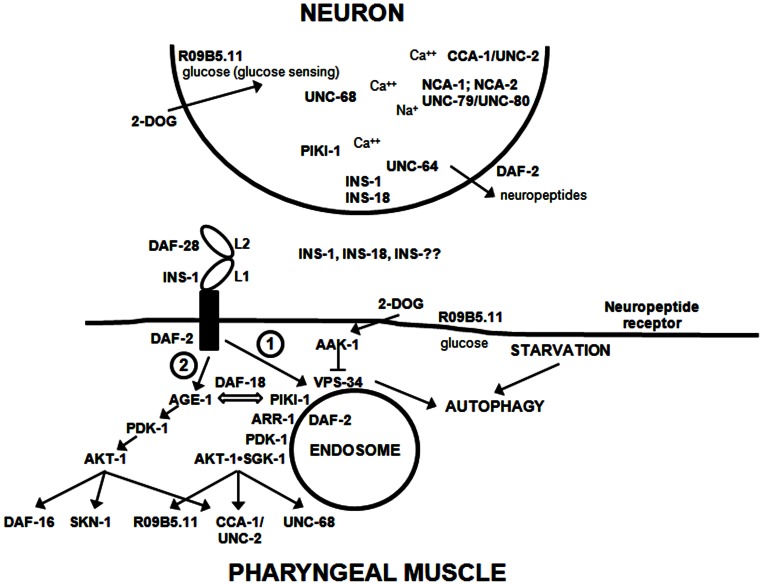
Model of how IIS affects recovery of pharyngeal pumping during starvation. This scheme is discussed in the text. Although the neuron is depicted as close by to the pharyngeal muscle, in reality, the neuronal signal (e.g., insulin and/or neuropeptides) may act more like a neurohormone over a greater distance. The depiction of the DAF-2 receptor is intended to show different binding modes for insulin molecules (e.g., INS-1 vs. DAF-28). Signaling downstream of DAF-2 bifurcates into Path 1, mediated by PIKI-1 and ARR-1, and Path 2, the classical pathway mediated by AGE-1 and AKT-1. INS-?? refers to additional *C. elegans* insulins that participate in this response.

We pursued this line of research based on the original observation that functional knockout of DAF-2 signaling in the *daf-2(e1370)* strain abolished recovery of pharyngeal pumping during food deprivation [38]. This phenotype of *daf-2* strains is not mediated by developmental effects because the animals have already reached the L4-young adult stage prior to brief (overnight) temperature shift to 25°C. As shown here, the degree of recovery generally showed a close correlation with the severity of defect in the *daf-2* allele. Thus, the class 2D alleles, *e979* and *e1391*, produced the most profound deficits in pumping recovery, whereas the *e1371* allele (class 1A) was essentially wild-type. The *daf-2(m579)* strain showed an intermediate phenotype, as expected for a class 2B allele. One exception to the trend was the *m596* allele, which is categorized as class 2A, but allowed normal recovery of pharyngeal pumping. The *e1391* and *e1370* alleles map to the tyrosine kinase domain of DAF-2, whereas *m579* and *e979* map to the L1 ligand-binding domain [60]. By contrast, *m596* is located in the L2 domain, and *e1371* is located in fibronectin-like III domain 2 [60]. We propose that *m579* and *e979* affect binding of all insulin molecules, whereas *m596* only affects the binding of a subset of insulins, which includes DAF-28 (and others with similar 3-D structural features), but not INS-1, INS-18 and structurally-related insulins. Thus, various insulin molecules bind to DAF-2, but with variation in the degree to which binding is stabilized by contacts with the L1 vs. L2 domains. We suggest that the different binding modes also transmit qualitatively distinct signals via the receptor. More specifically, there may be differential activation of class I vs. class II/III PI3Ks depending on the compartmentalization of downstream protein-membrane and protein-protein interactions (see Fig. 7). Our data suggest that additional insulin molecules besides INS-1 and INS-18 mediate recovery of pharyngeal pumping because the double mutant strain (*ins-1;ins-18*) is not as severely affected as strains (e.g., *e1391*) with major reductions in *daf-2* function. It is worth noting that both INS-1 and INS-18 are expressed in the pharynx [50], so they might produce direct (cell autonomous) effects on pharyngeal muscles.

In view of the insulin connection, we investigated the behavior of strains with mutations in genes linked to the release of insulin such as *unc-64* [61]. A consistent, yet preliminary, picture emerged from these studies. In *unc-64* strains, recovery of pharyngeal pumping was significantly reduced, as expected. Class II PI3Ks are also needed for glucose-stimulated insulin secretion [62], which would be consistent with the *piki-1* data and the effects of LY294002 reported here. In addition, the NALCN protein is expressed in beta cells of the pancreas and regulates insulin secretion [52]. A strain lacking both *C. elegans* orthologs, *nca-1;nca-2* [40], failed to recover normally, similar to strains lacking *unc-79* and *unc-80*, which are essential for functional expression of NALCNs. Additional evidence from our studies supports a functional role of NALCN in the recovery of pharyngeal pumping. First, a *gf* allele of *nca-1* [*unc-77(e625)*] showed accelerated recovery of pumping in response to food deprivation. Secondly, in behavioral assays, *unc-7* opposes the effect of mutations in *unc-79* and *unc-80* [40], [53], [56]. We found that the *unc-7(e5)* strain also recovered pharyngeal pumping much faster than wild-type animals, which would be consistent with its normal antagonism of *unc-79* and *unc-80*.

The ryanodine receptor (UNC-68) may either be upstream or downstream of DAF-2 in our system. It too regulates insulin secretion, especially in concert with elevated glucose concentrations [63]. However, ryanodine receptors enhance muscle contraction, and are also regulated by insulin [28], possibly via the potential Akt phosphorylation site described here. Previously, it has been noted that *unc-68* strains pump abnormally on food [64].

In addition, we showed that knocking out a *C. elegans* GLUT gene (*R09B5.11*) inhibited recovery of pharyngeal pumping. This is potentially consistent with a requirement for glucose to stimulate insulin secretion in *C. elegans*. Alternatively, glucose may be needed as a fuel source to sustain high rates of pharyngeal pumping. The fact that 2-DOG reduces pumping on food, and inhibits recovery of pumping off food, would be consistent with the latter interpretation. Once again, GLUTs could be upstream (controlling insulin release) or downstream of DAF-2.

These studies confirmed that kinases downstream of DAF-2, namely AKT-1 and SGK-1, were required for normal recovery of pharyngeal pumping. Potential targets of these kinases include various Ca^++^ channels [35], [36], and GLUT4 [65]. Loss of function of CCA-1, a T-type Ca^++^ channel, significantly impaired pumping recovery. Steger et al. [42] reported previously that *cca-1* mutants display electrophysiological abnormalities in pharyngeal muscle, and pump slightly slower on food. The mixed Ca^++^ channel inhibitor, mibefradil, further reduced pumping in the *cca-1* strain, which implicated additional Ca^++^ channels in the response. Moreover, flunarizine, a broad spectrum Ca^++^ channel antagonist, and mibefradil, a mixed-type inhibitor, produced the greatest inhibition of pumping, both on and off food, confirming the importance of Ca^++^ currents for normal pharyngeal pumping. Of course, CCA-1 and GLUTs can be regulated in other ways besides direct phosphorylation by Akt/SGK.

Perhaps, the most novel finding from our work concerns the role of PI3Ks in the IIS pathway during starvation. The PI3K inhibitor, LY294002, significantly reduced pharyngeal pumping on food and prevented recovery of pumping in the N2 strain in response to food deprivation. Surprisingly, LY294002 also inhibited basal pumping and pumping recovery in the *daf-16(m26);age-1(m333)* strain that lacks functional *age-1*, the only class I PI3K of *C. elegans*. LY294002 inhibits the class III PI3K, VPS-34, and class II PI3Ks, although PI3K-C2α is more resistant to inhibition [66], [67]. At the concentrations used in our studies (150 µM added to the plate), we anticipate that class II and class III PI3Ks will be inhibited. LY294002 has been reported to inhibit PI4K [68], so this is another possibility. However, several observations are more consistent with PI3Ks as the functional target in our system. (1) PIKI-1 (class II PI3K) is required for recovery of pharyngeal pumping. (2) The *daf-16(m26);age-1(m333)* strain actually shows accelerated recovery of pharyngeal pumping in the absence of LY294002, which suggests that *age-1* normally antagonizes the recovery response (see Fig. 7). (3) Loss of PTEN (DAF-18) also accelerates early recovery of pharyngeal pumping, which confirms an important role for 3-phosphorylated phosphoinositides in the response.

Class II and class III PI3Ks mainly synthesize PI(3)P with limited production of PI(3,4)P_2_ by the class II kinases [67], [69]. The synthesis of PI(3/3,4)P_/2_ is a key step in the signaling pathway that best accounts for the results reported here. We propose that signaling downstream of DAF-2 bifurcates along two pathways depending on accessory molecules, such as β-arrestin (ARR-1), and subcellular compartmentalization (see Fig. 7). Several groups have reported that insulin activates PI3K-C2α (PIKI-1 in *C. elegans*) in cell lines and muscle [70]–[72]. PI(3)P produced by PIKI-1 in response to insulin signaling and/or constitutively by VPS-34 will serve as bait to attract proteins with PX, FYVE, PH or other inositol lipid-binding domains to endocytic or endosomal membranes [73]. We note here for the first time (to our knowledge) that *C. elegans* SGK-1 has a PX domain similar to human SGK-3, formerly known as cytokine-independent survival kinase (CISK). This PX domain is likely to facilitate selective targeting of SGK-1 to endosomal membranes. We previously suggested that SGK-1 and AKT-1 form a signaling complex at the endosomes [74]. Here, we propose that the SGK-1•AKT-1 endosomal complex may phosphorylate different substrates than AKT-1 when it is situated at the cell surface or other locations in the cell (e.g., the nucleus). β-arrestin has also been reported to bind to 3-phosphorylated PI [75]. Furthermore, it acts in a signaling complex with insulin/IGF-1 receptors [33], [57].

The *daf-2* allele data suggest that distinct binding modes exist for different *C. elegans* insulin molecules, e.g., INS-1 vs. DAF-28. According to this model, INS-1and INS-18 act like antagonists in relation to DAF-28 because they down-regulate DAF-2 from the cell surface via association with ARR-1 and endocytosis. Interestingly, starvation for 24 hr reduces DAF-2 expression in *C. elegans* [76], which would be consistent with the β-arrestin pathway outlined in Fig. 7. On the other hand, INS-1 and INS-18 are agonists from the perspective of signals generated through Path1 (class II PI3K activation), which differs from the classical pathway (Path 2) activated by DAF-28 and related insulins.

An adaptive response to food deprivation appears to drive recovery of pharyngeal pumping. Regulation of this response may be achieved through several distinct mechanisms. Insulin (e.g., INS-1) is released by head neurons, such as AIA and ASI, in response to starvation [77], [78], and may increase pharyngeal pumping by up-regulating GLUTs, Ca^++^ channels and ryanodine receptors in muscle. In fact, INS-1 may be the unidentified molecule released from ASI neurons during dietary restriction that up-regulates energy metabolism [78]. The type of insulins produced in response to food deprivation may also tilt the balance of signaling downstream of DAF-2 toward activation of the PIKI-1-ARR-1-SGK-1 branch. Moreover, starvation would activate autophagy via VPS-34 in concert with PDK-1 and RSKS-1 (a p70 S6 kinase) [79], [80]. The significance of this pathway is underscored by the observation that 2-DOG and knockout of R09B5.11 inhibit recovery of pharyngeal pumping. These conditions would decrease glucose availability leading to activation of the 5′-AMP kinase, AAK-1, and inhibition of VPS-34 and autophagy [79]. Autophagy is required to maintain muscle mass and strength under normal conditions [81].

DMSO produced striking effects in our system, and the mechanisms are likely to be complex. On its own, DMSO modestly reduced pharyngeal pumping in all strains [except *pdk-1(mg142)*] on food, but completely abolished pumping and the recovery response of *daf-2(e1391)*. DMSO has been reported to directly inhibit insulin binding to its receptor [82], up-regulate PTEN and decrease Akt activity [83], and affect neurotransmission more generally [84], which would explain its modest adverse effects on pharyngeal pumping in the solvent control groups. The findings with *daf-2(1391)* suggest DMSO affects additional pathways besides IIS because DAF-2 signaling is largely blocked by shifting the temperature to 25°C. DMSO is not toxic under the conditions tested (0.8% concentration for a 24-hr exposure). Animals fully recover locomotion and pumping after removal from DMSO. Moreover, Wang et al. [85] found that DMSO at 0.5% and 2% concentrations significantly extended both mean and maximum lifespan in *C. elegans* by 25%. DMSO antagonism of insulin binding or DAF-2 signaling may explain its positive effects on lifespan in the N2 strain [85].

Our data begin to address some of the controversy in the literature concerning positive vs. negative effects of IIS down-regulation on muscle function. Previous studies have differed in their focus on partial vs. total loss of function in IIS pathways. For instance, Herndon et al. [86] reported that the long-lived *age-1(hx546)* strain maintains muscle integrity over time better than N2 controls; however, this strain still has partial IIS function. Moreover, groups have grown *daf-2* mutant strains at different temperatures (20°C vs. 25°C). At 25°C, DAF-2 signaling is essentially abolished, whereas at 20°C it is merely reduced. Huang et al. [47] reported preservation of fast pumping in *daf-2(e1370)* with aging, but the animals were grown at 20°C. Chow et al. [46] observed that *daf-2(e1370)* animals dramatically reduced pharyngeal pumping when grown at 25°C, but rebounded when shifted back to 20°C. While there was less tissue damage in the pharyngeal terminal bulbs of *daf-2(e1370)* adults with low pumping rates, there was also significant thinning and distortion of muscle tissue in the isthmuses of these same animals. Intriguingly, the authors speculated that this muscle loss may be the result of autophagy, which fits with the model presented here.

There are additional paradoxes to explain. IIS is anabolic for muscle and maintains muscle mass, yet a moderate reduction in IIS extends lifespan. Furthermore, when IIS is completely abolished, half of the animals die more quickly than normal, whereas the other half lives longer [4], [5], [13]. The scheme in Fig. 7 may help to resolve these paradoxes. Depending on the context of signaling, class I or class II PI3Ks will be activated downstream of the insulin/IGF-1 receptor producing different outcomes. Classical PI3K signaling (Path 2) will activate Akt leading to phosphorylation of targets such as DAF-16 (FOXO), SKN-1, GLUTs, and GSK-3. This pathway will regulate growth, development, hypoxia resistance and lifespan largely via genomic effects. By contrast, when the class II PI3K arm is activated (in concert with β-arrestin), different targets are affected, including SGK-1 and Ca^++^ channels via acute, non-genomic effects (e.g., trafficking). Furthermore, class I PI3Ks antagonize certain actions of classII/III PI3Ks (60), which may partly explain why the *daf-16(m26);age-1(m333)* null strain shows quicker recovery of pharyngeal pumping than controls. We imagine that the bias in these pathways is determined by the nature of insulin binding to its receptor (e.g., INS-1 vs. DAF-28), the state of the receptor itself (e.g., complexed with co-activating molecules such as β-arrestin), and/or the cellular location of receptor complexes.

Our data potentially shed light on another aspect of the paradox: even within a signaling pathway chronic over-activity may be harmful. Thus, the *akt-1(gf)* strain recovers pumping more quickly for the first 6 hr, but then reverses direction after 24 hr of starvation. The *daf-18* strain behaves similarly, presumably because the mutation prevents turnover of PI(3,4,5)P_3_, thereby prolonging activation of Akt. Excessive Akt activity would inhibit autophagy, tilt the balance away from Path1 (Fig. 7), and compromise cell viability [87]. Cohen and Dillin [88] astutely observed that organisms normally maintain optimum levels of IIS, and concluded that “IIS levels either higher or lower than the optimal rate interfere with metabolism, can cause disease and eventually shorten lifespan.” Along these same lines, Kang et al. [89] showed that too much or too little autophagy during starvation adversely affects pharyngeal pumping and viability in *C. elegans*. They found that this response is regulated, in part, by muscarinic cholinergic receptors, which also control insulin release by β-cells of the pancreas [52], and perhaps pharyngeal muscle cells in our system.

Curiously, the *pdk-1(gf)* strain sustains high rates of pharyngeal pumping off food, even after 24 hr of food deprivation. This observation agrees with the work of Szewczyk et al. [90], which showed that the *pdk-1(gf)* mutation suppressed protein degradation in muscle and protected against the adverse effects of LY294002. In contrast to our work, the latter studies examined the effects of *pdk-1(mg142)* in well-fed animals. We suspect that the response in our system involves activation of additional PDK-1 targets (e.g., RSKS-1 [pS6K]) as part of a nutrient-sensing response. PDK-1 regulates contractility in cardiac muscle and prevents heart failure [91], which is consistent with its ability to sustain rapid pharyngeal pumping during starvation. Altogether, the studies reported here highlight the importance of IIS in promoting muscle performance, and encourage future work on the connections between IIS, nutritional status and sarcopenia in aging.
